# The metabolomic plasma profile of patients with Duchenne muscular dystrophy: providing new evidence for its pathogenesis

**DOI:** 10.1186/s13023-023-02885-1

**Published:** 2023-09-05

**Authors:** Huayan Xu, Xiaotang Cai, Ke Xu, Qihong Wu, Bei Xu

**Affiliations:** 1 Department of Radiology, Key Laboratory of Birth Defects and Related Diseases of Women and Children of Ministry of Education, West China Second University Hospital, Sichuan University, Chengdu, Sichuan China; 2 Department of Rehabilitation Medicine, West China Second University Hospital, Sichuan University, Chengdu, Sichuan China; 3 Department of Clinical Laboratory, School of Medicine, Mianyang Central Hospital, University of Electronic Science and Technology of China, Mianyang, Sichuan China; 4 Department of Critical Care Medicine, Frontiers Science Center for Disease-related Molecular Network, State Key Laboratory of Biotherapy, West China Hospital, Sichuan University, Chengdu, Sichuan China

**Keywords:** Duchenne muscular dystrophy, Metabonomics, Mass spectrometry, Plasma

## Abstract

**Background:**

Duchenne muscular dystrophy (DMD) is a fatal genetic muscle-wasting disease that affects 1 in 5000 male births with no current cure. Despite great progress has been made in the research of DMD, its underlying pathological mechanism based on the metabolomics is still worthy of further study. Therefore, it is necessary to gain a deeper understanding of the mechanisms or pathogenesis underlying DMD, which may reveal potential therapeutic targets and/or biomarkers.

**Results:**

Plasma samples from 42 patients with DMD from a natural history study and 40 age-matched healthy volunteers were subjected to a liquid chromatography-mass spectrometry-based non-targeted metabolomics approach. Acquired metabolic data were evaluated by principal component analysis, partial least squares-discriminant analysis, and metabolic pathway analysis to explore distinctive metabolic patterns in patients with DMD. Differentially expressed metabolites were identified using publicly available and integrated databases. By comparing the DMD and healthy control groups, 25 differential metabolites were detected, including amino acids, unsaturated fatty acids, carnitine, lipids, and metabolites related to the gut microbiota. Correspondingly, linoleic acid metabolism, D-glutamine and D-glutamate metabolism, glycerophospholipid metabolism, and alanine, aspartate, and glutamate metabolism were significantly altered in patients with DMD, compared with those of healthy volunteers.

**Conclusions:**

Our study demonstrated the abnormal metabolism of amino acids, energy, and lipids in patients with DMD, consistent with pathological features, such as recurrent muscle necrosis and regeneration, interstitial fibrosis, and fat replacement. Additionally, we found that metabolites of intestinal flora were disordered in DMD patients, providing support for treatment of intestinal microbia disturbance in DMD diseases. Our study provides a new research strategy for understanding the pathogenesis of DMD.

## Background

Duchenne muscular dystrophy (DMD) is the most common inherited form of muscular dystrophy, causing progressive muscle degeneration and weakness that culminates in respiratory failure and premature death [1]. According to reports from the USA and UK, the prevalence of DMD is respectively 15.9 cases and 19.5 cases per 100,000 live male births, respectively [2, 3]. In people with DMD, an X-linked mutation interferes with the ability to produce functional dystrophin in the muscles [4]. Absence of dystrophin cause muscle fragility and loss of muscle fibers, and replaced by fat and fibrosis [5].

The DMD disease manifests through compromised membrane integrity, abnormal calcium homeostasis, chronic inflammation, and fibrosis, as well as impaired tissue remodeling [6]. Although separate efforts targeting these pathways, including the use of calcium channel blockers to restore calcium homeostasis and anabolic steroids to restore muscle mass, have been clinically tested, they have not yielded any clinical benefits associated with the overall course of the disease [7]. Glucocorticoids are currently the only medications recognized to benefit DMD based on treatment guidelines, but their efficacy is suboptimal with considerable side effects [8]. To date, there is no cure for DMD, and the few treatments available do not dramatically delay disease progression. This illustrates the need for a better understanding of the underlying mechanisms involved in the disease process, which may elucidate potential therapeutic targets and/or biomarkers.

Metabolic deficits had been found dystrophic skeletal muscle, and may have promotional effect in the disease progression [9]. One previous study showed that lipid metabolism might be a critical metabolic disturbance in DMD, and studies regarding the skeletal muscle of mdx mice (a genetic model of DMD) uncovered dysregulation of cholesterol and fatty acid metabolism transcription factors (SREBP-1 and SREBP-2), disruption of the mevalonate pathway, and accumulation of cholesterol in dystrophic muscles [10]. Another study revealed DMD muscle metabolic remodeling patterns using ultra-high resolution mass spectrometry [11]. Compared to control biopsies, Duchenne biopsies showed a decrease in 7 metabolites, including adenosine triphosphate and glycerophosphate choline, and an increase in 27 metabolites, including sphingomyelin, phosphatidylcholine, phosphatidic acid, and phosphatidylserine. In addition, xanthine oxidase activity has been as a contributing factor in mdx mouse [12]. Most of these dysregulated metabolites have a strong connection to energy and phospholipid metabolism, revealing the profound metabolic remodeling of phospholipids and energy metabolism in DMD. Further, metabolic supplement therapy for DMD has been shown to be effective in some pre-clincal or clinical studies. In mice, adenylosuccinic acid, a purine nucleotide cycle metabolite, significantly improved the histopathological features of DMD by reducing damage area, the number of centronucleated fibres, connective tissue infiltration of mdx tibialis anterior [13]. Recently, a randomized clinical trial for DMD patients, evaluating the effects of combination of I-citrulline and metformin treatment. Compared with monotherapy, a reduction in motor function decline was observed among the stable subgroup of patients treated with combination therapy [14]. Despite great progress has been made, its underlying pathological mechanism is still worthy of further study, in order to discover more valuable therapeutic targets for DMD disease.

Metabolomics is an omics technique that allows exploration of the metabolome by comprehensive measurement of metabolite levels in a biological sample [15]. Metabolites are small molecular mass components or metabolic intermediates that can be detected in biological fluids and tissues. They regulate and maintain physiological homeostasis and have many biological functions [11, 16]. At present, only one study has tested metabolomics to identify plasma metabolic changes between the DMD and healthy control (HC) groups. Simina et al. found that arginine, creatine, creatinine, and androgen derivatives were dramatically altered in patients with DMD, and the creatinine-to-creatinine ratio, which has been used as a biomarker of DMD disease in a previous clinical trial, was significantly associated with disease progression, but not specific for detailed metabolism disorders [16, 17]. Overall, DMD is relatively understudied with regard to circulating metabolites and metabolic pathways.

Metabolomic studies utilize mass spectrometry because of its good detection sensitivity, broad coverage of metabolomes, and rapid data acquisition turnaround [18]. Therefore, in this study, ultra-high performance liquid chromatography-tandem mass spectrometry (UPLC-MS/MS) technology was used to study the metabolomics of the plasma of patients with DMD. Their pathogenesis is discussed and analyzed at the metabolic level to provide a reference for exploring new circulating biomarkers or therapeutic targets for DMD.

## Methods

### Study design and participants

Between July 2018 and January 2021, 42 patients with DMD were prospectively recruited for this study, which was approved by the Institutional Review Board (IRB) of Sichuan University (K2019056). Genetic testing and/or skeletal muscle pathology confirmed DMD diagnosis in all cases. Additionally, 40 healthy volunteers without any known chronic or major illness and without any treatment were recruited as the HC group. The participants in the study were all male, and the cases and controls were age matched. Clinical data such as routine drug usage, creatine kinase, loss of ambulatory and North Star Ambulatory Assessment score was recorded.

### Sample collection and preparation for metabolomics

All plasma samples were collected after overnight fasting. Sterile EDTA-K2 anticoagulation BD vacuum blood collection tubes were used to obtain whole blood samples (5 mL). At room temperature, the tubes were gently shaken and centrifuged at 3000 rpm for 10 min. The supernatant (plasma) was aspirated into 1.5-mL microfuge tubes and stored at -80 °C for analysis.

After thawing on ice, 100 µL of plasma was mixed with 300 µL of methanol: acetonitrile (1:1 v/v) solution to extract a variety of small molecular metabolites. After vortexing, sonication, and incubation at -20 °C for 30 min, the supernatants were filtered and transferred to sample bottles with a 0.22-µm microporous membrane. Aliquots of all plasma samples (10 µL) were pooled for quality control (QC) and system adjustment. The QC samples were treated in a similar manner to that of the analytical samples.

### Metabolite detection

An ultra-performance liquid chromatography (UPLC) system (Agilent1290 Infinity II; Agilent Technologies Inc., CA, USA) connected to a high-resolution tandem mass spectrometer (TripleTOF 5600 Plus; AB SCIEX, Framingham, MA, USA) was used to conduct the metabolomic analysis. Reversed-phase separation was performed on an ACQUITY HSS T3 column (100 × 2.1 mm, i.d. 1.8 μm; Waters, Milford, USA). The mobile phase composition was determined using a gradient elution of solvent A (0.1% formic acid in water) and solvent B (0.1% formic acid in acetonitrile), as previously described [19]. The flow rate was constant at 0.30 mL/min, and the column temperature was set at 30 °C.

Independent data acquisition (IDA)-based auto-MS2 mode, coupled with full scan mode, was used to acquire mass spectrometry data. These parameters were set for mass spectrometry: m/z range, 80-1000; declustering potential, 80 V (+) and − 80 V (-); collision energy, 10 V (+) and − 10 V (-); ion spray voltage floating, 5500 V (+) and − 4500 V (-); interface heater temperature, 550 ºC; curtain gasl 35 psi; and ion source gas 1/ion source gas, 2:55 psi. In both positive and negative modes, the m/z range of IDA analysis was set at 50-1000. The positive and negative ion modes had collision energies of 35 V and − 35 V, respectively, and the collision energy spread was 15 V.

For every six samples, the mass accuracy was calibrated throughout the study. Additionally, to evaluate the reliability of the large-scale metabolomics analysis, QCs were introduced after every 10 samples.

### Metabolomics analysis and annotation

Ultra-high performance liquid chromatography tandem mass spectrometry (UPLC-MS/MS) raw data were acquired and processed using Analyst TF software (version 1.7.1, AB SCIEX). As part of the metabolomics workflow, peaks were selected, quality was assessed, missing values were imputation, normalization, transformation, and scaling were conducted.

For accurate metabolite characterization, the processed molecular weights of the metabolites were validated, matched, and annotated using the standard database and custom databases, including METLIN (http://metlin.scripps.edu/), Kyoto Encyclopedia of Genes and Genomes (KEGG) (http://www.kegg.jp/kegg/pathway.html), LipidMaps (https://www.lipidmaps.org/), Human Metabolome Database (HMDB) (https://hmdb.ca/), MassBank (https://massbank.eu/), and PubChem Database (https://pubchem.ncbi.nlm.nih.gov/).

A multivariate analysis with SIMCA 15.0.2 (Umetrics AB, Umea, Sweden) was conducted. LC-MS/MS data was used to perform an unsupervised, non-targeted principal component analysis (PCA) to visualize holistic variation and to track stability over time. Partial least-squares discriminant analysis (PLS-DA) was used to identify significant metabolites. Interpretability and predictability were measured using R2 and Q2 to test the validity of the model and prevent overfitting. With PLS-DA, variable importance in projection (VIP) was computed. One-dimensional analyses were performed with a paired Student’s t-test to determine the P-values. A t-test was used in conjunction with the PLS-DA method to assess differences in metabolites between groups (with VIP > 1 and P < 0.05).

### Statistical analysis

The statistical analyses were done using SPSS 25.0 (International Business Machines Corp., Armonk, NY, USA). Calculations of normally distributed data were reported as mean and standard deviation. Among multiple groups with homogeneous variance, an analysis of variance (ANOVA) was used followed by a least significant difference (LSD) t-test; otherwise, Welch’s t-test and Dunnett’s T3 test were adopted. Statistical data with a non-normal distribution were calculated as the median (interquartile range) (M [P25, P75]). Comparisons between groups were made using the Kruskal-Wallis test. Comparing count data among the groups was carried out using the Chi-square test. The level of statistical significance was set at a P-value of 0.05.

## Results

### Population and clinical characteristics

A summary of the characteristics of the study participants is presented in Table 1. We found no significant demographic differences between the two groups including individual BMI values, except that DMD patients had lower body weight values than healthy controls. Almost half of DMD patients (22/42) take corticosteroid medications, and some DMD received vitamin (30/42) and calcium supplements (30/42). Creatine kinase was obviously increased in DMD. Only 4 DMD patients had loss of ambulatory. North Star Ambulatory Assessment score was 10.9 ± 2.00, which is lower than normal score 34.

**Table 1:** Baseline characteristics of healthy and DMD children

	Healthy Control (n = 40)	DMD Patients (n = 42)	t/χ2, *P*
Age, years	9.96 ± 2.79	9.63 ± 2.23	0.583, 0.124
Height, cm	133.00 ± 12.18	127.57 ± 11.93	1.989, 0.174
Weight, kg	32.50(27.90, 37.00)	27.40(24.00, 35.00) *	2.244, 0.025
BMI, kg/m^2^	19.057 ± 2.885	18.577 ± 3.653	1.547, 0.093
*Corticosteroids (n/n)*	0/40	22/42	**-**
*Vitamin D supplement (n/n)*	22/40	30/42	**-**
*Calcium supplement (n/n)*	10/40	30/42	**-**
*Creatine kinase (normal range 39-192U/L)*	**-**	11115.79 ± 1735.99	**-**
*Loss of ambulatory*	**-**	4/40	**-**
*North Star Ambulatory Assessment score*	**-**	10.9 ± 2.00	**-**

BMI: body mass index; *: Compared with HC group, *P* < 0.05

### Multivariate statistical analysis of metabolites

Liquid chromatography-mass spectrometry often serve as analytical methods for analyzing metabolomic data and measuring mass-to-charge ratios. The retention time, exact mass, and peak intensity of the DMD and HC groups were analyzed using multivariate statistical methods.

**Fig. 1:**
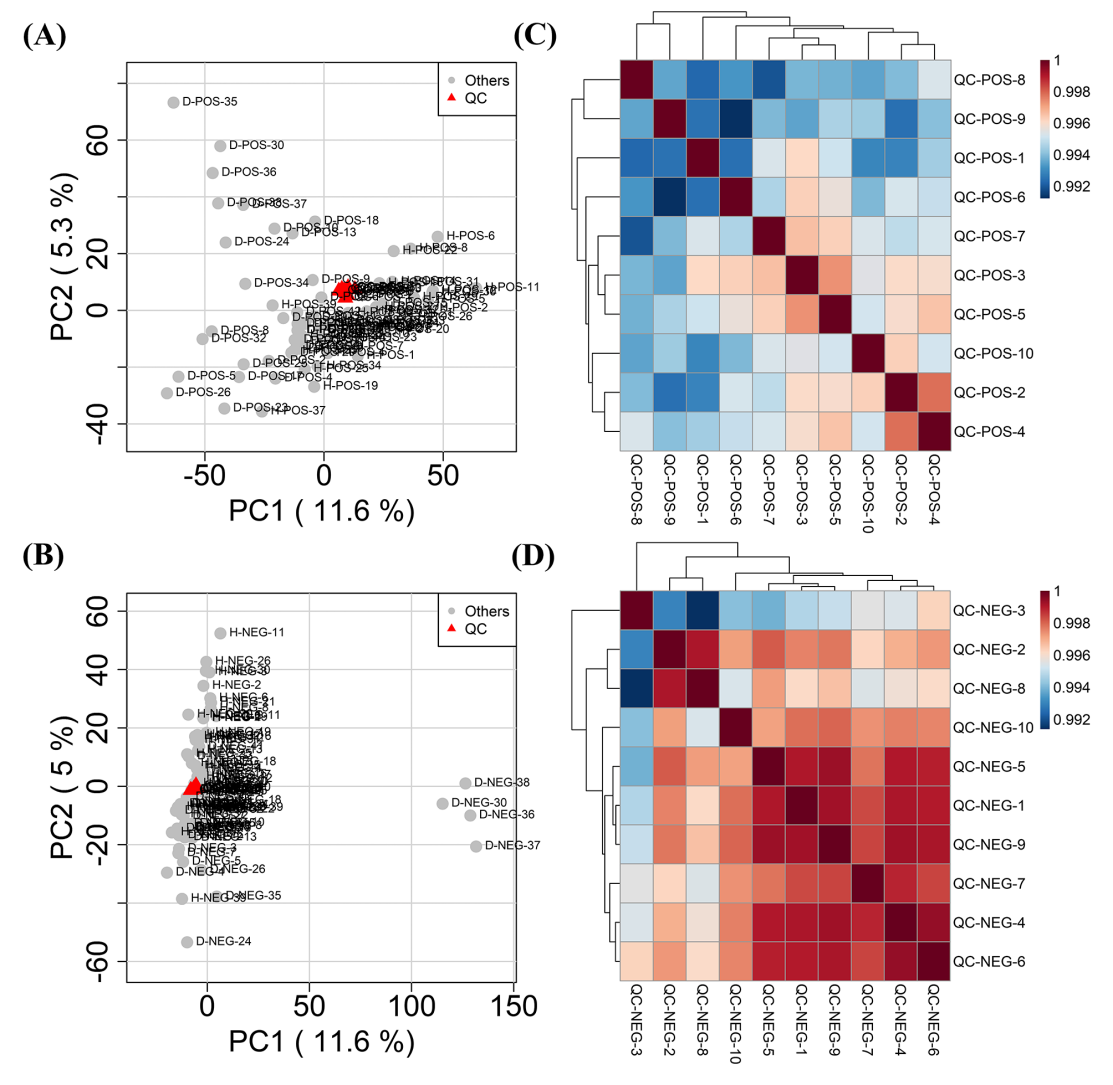
PCA score plots and correlation analysis of QC samples in ESI+ (**A, C**) and ESI− (**B, D**) scan modes

**Fig. 2:**
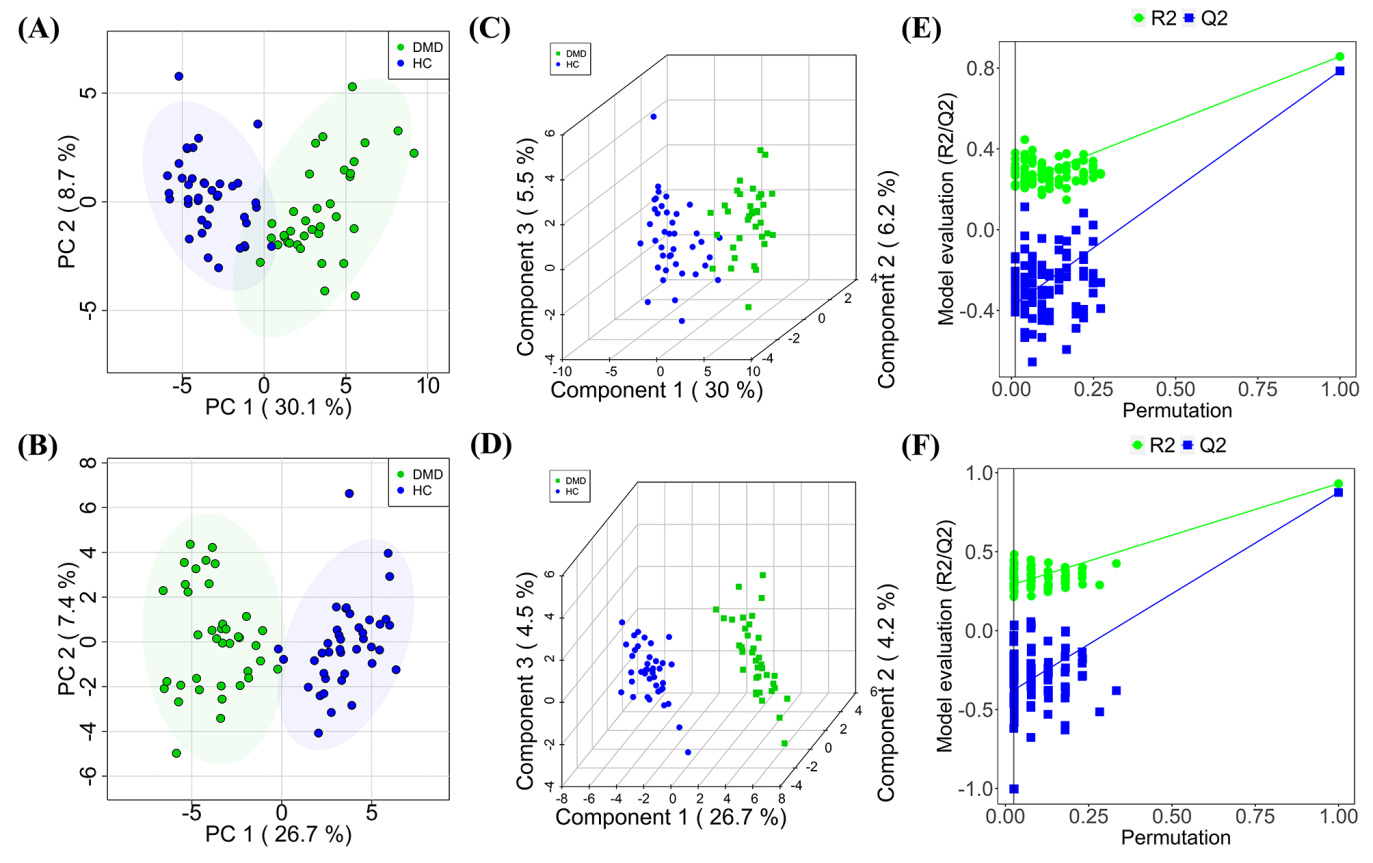
Plots of PCA (**A, B**) and PLS-DA scores (**C, D**) with permutation testing (**E, F**) for healthy controls and DMD patients comparison in the ESI + and ESI − scan modes

Unsupervised PCA analysis was conducted. Through PCA, highly correlated metabolic features are reduced to a smaller set of principal components. This superiority enables PCA score plots to visualize the pattern described by the model which can be used to identify batch effects. In this study, the PCA plot of QC samples showed clustering (Fig. 1A and B), and correlation heatmaps showed strong correlations (Fig. 1C and D). The results indicated that the analytical system was stable and repeatable. Moreover, in two-dimensional (2D) plots, participants with DMD were distinguished from those in the HC group (Fig. 2A and B), revealing metabolic pattern defects in patients with DMD.

We applied PLS-DA to obtain an in-depth understanding of the different metabolic profiles and unravel potential biomarkers with better discriminative power than PCA. The DMD and HC comparison showed distinct clustering for both the positive and negative ion modes, demonstrating an explicit separation of the two participant cohorts (Fig. 2C and D). R2X, R2Y, and Q2 (cumulative) parameters are used in the evaluation of the PLS-DA model. They were established as 0.36, 0.86, and 0.79 in the positive mode and 0.41, 0.96, and 0.89 in the negative mode, respectively. The high Q2 values of the PLS-DA model demonstrated high accuracy. The permutations of 200 random variables were performed to determine whether the supervised PLS-DA models were overfitted. PLS-DA was reliable because its Q2 distribution had Y-intercepts below zero for both positive and negative ions (Fig. 2E F). The DMD and HC groups were distinguished by PCA and PLS-DA models with highly effective metabolite identification.

### Differential metabolite analysis and identification

**Fig. 3:**
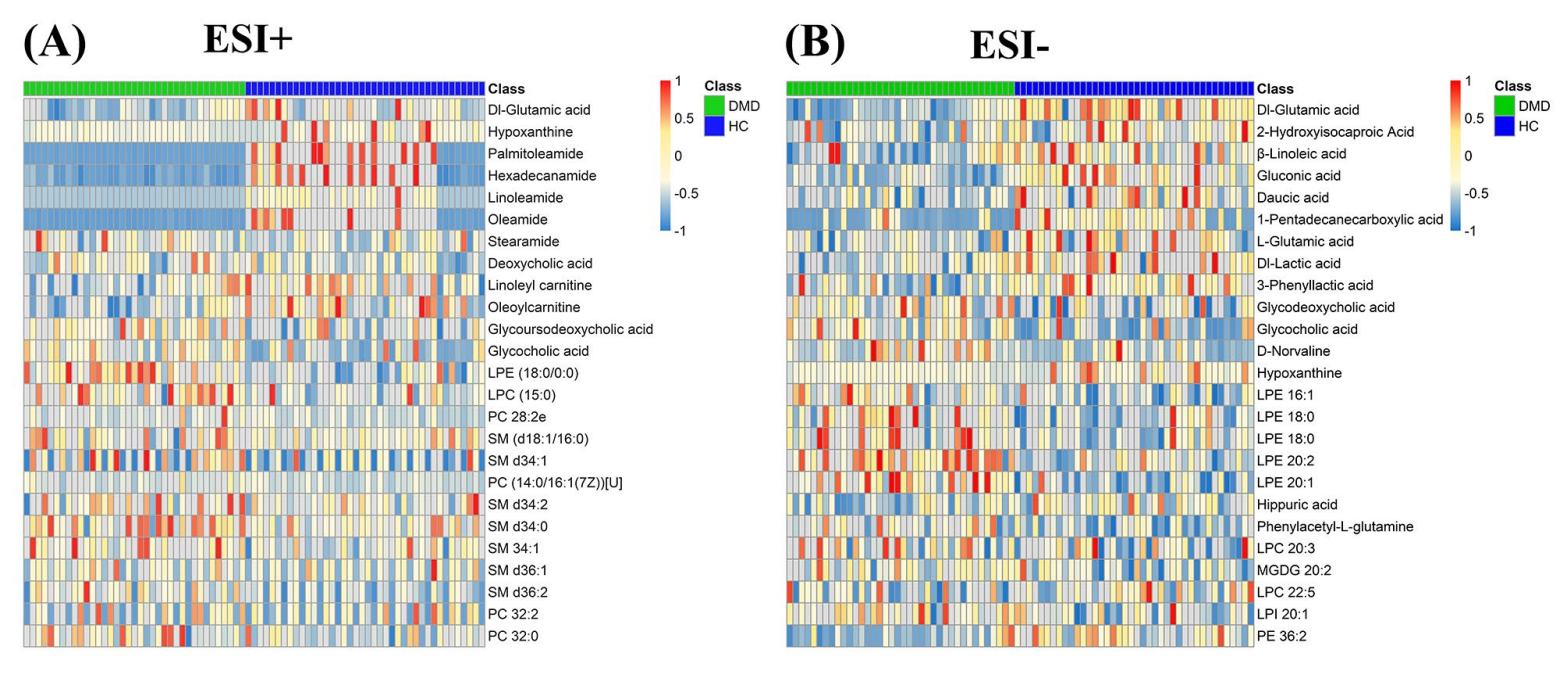
Differential metabolite heat maps in ESI+ (**A**) and ESI− (**B**) scan modes. The columns represent samples, the rows represent metabolites, and the relative content of the metabolites is displayed by color

Following the alignment of peaks and elimination of missing values, we observed 6207 and 6539 peaks in the ESI + and ESI- modes using the MS/MS data, respectively. Qualitative identification was conducted using publicly available and integrated databases, and 518 and 501 metabolites were identified using the positive and negative ion modes, respectively. Next, 87 different metabolites with a fold-change threshold of > 1.5 or < 2/3, VIP of > 1, and Student’s t-test threshold of P < 0.05 were selected for the comparison of DMD and HC. Heat maps of 25 representative differential metabolites showed clear clustering between the DMD and HC groups in both positive and negative modes (Fig. 3), consistent with the PLS-DA results.

**Fig. 4:**
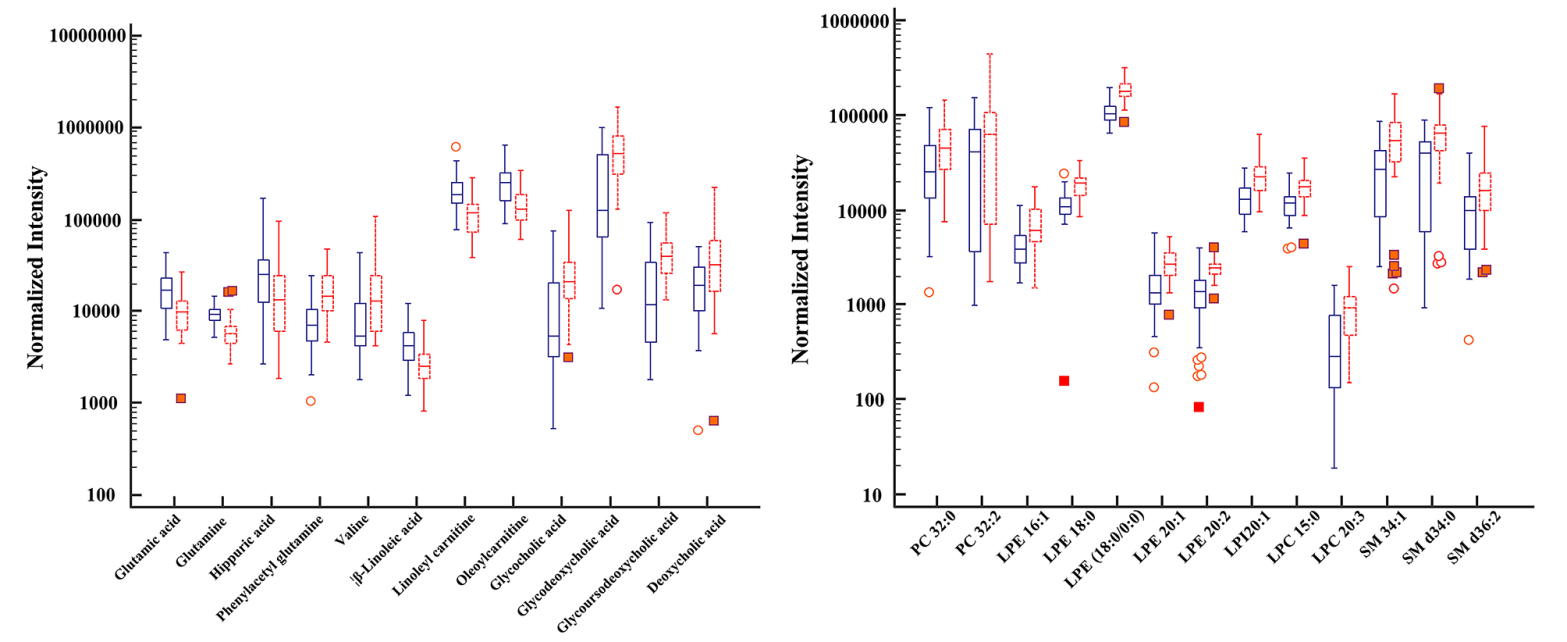
The box plot of normalized intensity peak areas of significantly changed metabolites in DMD group when compared with HC group. Red represent the DMD group, and blue represent the HC group

The most abundant classes of metabolites were amino acids, unsaturated fatty acids, carnitine, bile acids, and lipids (Table 2). Screening criteria (FC of > 1.5 or < 2/3, VIP > 1, and P < 0.05) identified 25 significantly altered molecules. These differential metabolites are semi-quantitatively analyzed in Fig. 4. In patients with DMD, the levels of glutamic acid, glutamine, hippuric acid, β-linolenic acid, linoleic acid carnitine, and oleoyl carnitine decreased, whereas the levels of valine; phenylacetyl-L-glutamine; glycine cholic acid; glycine deoxycholic acid; glycine ursodeoxycholic acid; deoxycholic acid; and most lipids, such as phosphatidylcholines (PC), lysophosphatidylethanolamines (LPE), and sphingomyelin (SM) increased.

**Table 2:** List of statistically significant metabolites in DMD vs. HC comparisons

Metabolites		Scan mode	Rt (s)	m/z	Adducts	DMD vs. HC			
						Log2(FC)	*P* (T test)	VIP	Trends
Amino acids	Glutamic acid	ESI-	73.431	128.036	M-H_2_O-H	-1.181	< 0.001	1.955	↓
	Glutamine	ESI+	47.218	146.080	M + H	-1.072	< 0.001	1.857	↓
	Hippuric acid	ESI-	300.935	178.051	M-H	-0.802	0.021	1.009	↓
	Phenylacetyl-glutamine	ESI-	348.896	527.213	2 M-H	1.143	< 0.001	2.008	↑
	Valine	ESI-	420.136	293.176	2 M + Hac-H	1.072	0.005	1.263	↑
Unsaturated fatty acids	β-Linolenic acid	ESI-	419.308	279.232	M-H	-0.766	0.0001	1.582	↓
Carnitine	Linoleyl carnitine	ESI+	435.747	424.342	M + H	-0.784	< 0.001	1.536	↓
	Oleoylcarnitine	ESI+	461.927	426.358	M + H	-0.766	< 0.001	1.635	↓
Bile acids	Glycocholic acid	ESI-	372.288	464.302	M-H	1.121	0.005	1.122	↑
	Glycodeoxycholic acid	ESI-	406.477	448.307	M-H	0.950	0.0004	1.460	↑
	Glycoursodeoxycholic acid	ESI+	406.026	450.322	M + H	1.031	< 0.001	1.543	↑
	Deoxycholic acid	ESI+	462.698	357.279	M + H-2H_2_O	1.174	0.002	1.323	↑
Lipids	PC 32:0	ESI+	1117.300	756.555	M + Na	0.712	0.006	1.058	↑
	PC 32:2	ESI+	730.026	730.539	M + H	0.872	0.029	1.018	↑
	LPE 18:0	ESI-	552.975	480.310	M-H	0.655	< 0.001	2.289	↑
	LPE 20:1	ESI-	590.366	506.326	M-H	0.748	< 0.001	1.656	↑
	LPE 20:2	ESI-	526.317	504.311	M-H	0.856	< 0.001	2.269	↑
	LPE (18:0/0:0)	ESI+	574.100	482.324	M + H	0.785	< 0.001	2.489	↑
	LPI 20:1	ESI-	386.676	625.361	M-H	0.799	< 0.001	1.997	↑
	LPE 16:1	ESI-	444.569	450.263	M-H	0.803	< 0.001	1.733	↑
	LPC 20:3	ESI-	405.790	590.235	M + FA-H	0.976	< 0.001	1.525	↑
	LPC 15:0	ESI+	487.913	504.309	M + Na	0.634	< 0.001	1.675	↑
	SM d34:0	ESI+	1118.730	705.591	M + H	0.851	< 0.001	1.360	↑
	SM d36:2	ESI+	812.360	729.590	M + H	0.983	0.002	1.203	↑
	SM 34:1	ESI+	729.672	725.557	M + Na	1.034	< 0.001	1.383	↑

“↑”: Compared with HC group, the differential metabolites were significantly increased in DMD group

“↓”: Compared with HC group, the differential metabolites were significantly decreased in DMD group

### Perturbed pathways identified in group comparisons

**Fig. 5:**
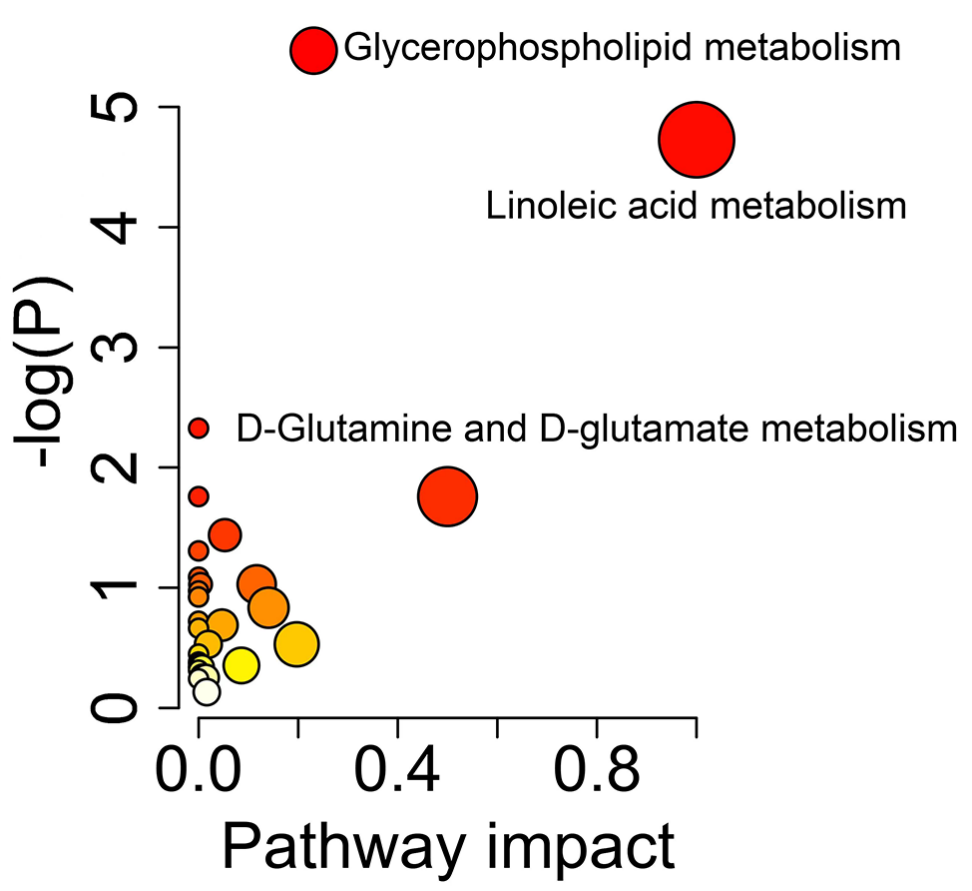
Bubble diagram of metabolic pathways between DMD and HC groups

The metabolic pathways associated with differential metabolites were examined. In the analysis, the accumulative percentages of importance for the matched metabolite nodes were calculated, and permutation-based P-values were computed and corrected for multiple testing to produce a permutation-based false-discovery rate (FDR) (-log [P] value). Based on the pathway impact score and –log (P) value, the perturbed pathways were mainly enriched in linoleic acid metabolism; D-glutamine and D-glutamate metabolism; glycerophospholipid metabolism; and alanine, aspartate, and glutamate metabolism (Fig. 5; Table 3).

**Table 3:** Significantly altered metabolic pathways between DMD and HC groups

Pathway name	KEGG.id	-log(P)	Impact	Hits
Linoleic acid metabolism	Hsa00591	4.73	1	2
D-Glutamine and D-glutamate metabolism	Has00471	1.76	0.5	1
Glycerophospholipid metabolism	Hsa00564	5.47	0.23	5
Alanine, aspartate and glutamate metabolism	Hsa00250	0.53	0.20	1

## Discussion

Dystrophic tissues undergo progressive metabolic changes, which is not only due to the obesity or metabolic syndrome at the later disease stage but also disease itself. Metabolism disorder may be a driving factor of disease progression of this inherited disease [9]. Previous researches had reported various metabolic defects are existed in the dystrophin-deficient skeletal muscle [10] and the changes of metabolites associated closely with the disease progression. Recent years have seen an increased interest in metabolomics because of the technique improvement and its auxiliary role in identifying metabolic mechanisms in organisms. More detailed information will be acquired by the metabolomics analysis. Unfortunately, it has been less extensively studied for investigating the circulating metabolic disorders of DMD compared with an HC group. The analysis of plasma metabolites in patients with DMD enables us to better understand the underlying mechanisms of DMD from the perspective of circulation metabolism and thus provides potential therapeutic targets.

Our study used LC-MS profiling to discover novel metabolic biomarkers and pathways for DMD in the plasma of 42 patients with DMD and 40 age-matched controls. A total of 25 representative molecular biomarkers differed significantly between patients with DMD and HCs. These metabolites include amino acids, unsaturated fatty acids, carnitine, lipids, and other metabolites related to the gut microbiota. Correspondingly, the metabolic pathways that were significantly altered were linoleic acid metabolism; D-glutamine and D-glutamate metabolism; glycerophospholipid metabolism; and alanine, aspartate, and glutamate metabolism.

Glutamate and glutamine are essential for nitrogen metabolism, conveying amino groups from ammonia to the liver in a nontoxic manner, and providing amino groups for several biomolecule synthesis including peptides and nucleotides [20]. A decrease in glutamate and glutamine levels was observed in patients with DMD and was mainly correlated with protein replacement requirement due to the intense muscle regeneration (mainly in limb muscles) which had been observed in mdx mice, or with higher peptides consumption as an energetic source in dystrophic muscles to compensate for energetic deficits [21]. Fibrosis is one of the pathological features of DMD, and previous studies have found that glutaminolysis plays an important role in fibrosis [22]. Enhanced conversion of glutamine to glutamate confers resistance to apoptosis and promotes stabilization of collagen via mTOR signaling [23, 24]. The first key enzymatic step in glutamine breakdown is the conversion of glutamine to glutamate by glutaminases [25]. The increased consumption of extracellular glutamate by myofibroblasts results in a decrease in plasma glutamate content, which is consistent with the findings of this study. In vivo, bleomycin-induced lung fibrosis and TGFβ1 can be ameliorated by the inhibition of glutaminase 1 [26, 27]. Therefore, we hypothesized that targeting glutaminolysis might be one way to inhibit muscle fibrosis in DMD.

The fatty acid metabolism pathway is dysregulated in DMD, and we observed an increase in plasma valine levels. The derivative 3-hydroxyisobutyrate, generated during valine metabolism, can activate fatty acid transport in endothelial cells, increase fatty acid uptake in muscle cells, and promote muscle lipid accumulation [28]. This finding may explain the pathological changes that lipid droplet synthesis also appeared to increase in DMD in previous researches [29]. This may be one of the reasons for enhanced muscle fat replacement in patients with DMD and may also be a therapeutic target. In addition, our study found that the levels of unsaturated fatty acids β-linolenic acid, linoleate carnitine, and oleylcarnitine in DMD were reduced, indicating impaired energy metabolism in DMD [30, 31], and we speculated that this might be related to muscle cell necrosis. Since fatty acids oxidation mainly participates in energy metabolism, downregulated fatty acids oxidation may induce fibrosis through the energy metabolism disorders [32]. Previous studies have found that intracellular fatty acid oxidation is also downregulated in renal tubulointerstitial fibrosis in mice and humans and that its restoration protects against fibrosis [26]. Fatty acid oxidation is downregulated in patients with DMD [17], suggesting that it may also be related to the development of muscle fibrosis in DMD, and provided a new research direction of fibrosis of DMD.

Fat replacement is a pathological feature of DMD and is accompanied by abnormal fat metabolism [33]. Disorders of phospholipid metabolism have been previously reported in patients with DMD [11]. Dabaj et al. found that PC, lysophosphatidylcholine (LPC), phosphatidic acid (PA), phosphatidylserine (PS), and SM classes, as well as triacylglycerols, are increased in DMD muscles compared to control muscles. DMD plasma also contains elevated levels of PC, LPE, LPC, and SM. Because DMD is characterized by glycerophosphocholine deficiency [34], an explanation for this metabolic remodeling could be the decline in lysophospholipase activity, which catalyzes lysophosphatidylcholine deacylation and contributes to glycerophosphocholine production [35]. Phosphatidylcholine can accumulate and be redirected to other metabolic pathways in response to this impairment, with increases in PA, SM, phosphatidylserine, and triacylglycerol [11]. It has been reported that changes in Ca2 + homeostasis and phospholipid homeostasis in DMD may lead to mitochondrial membrane fragility and morphological changes, interfering with energy and oxidative metabolism; thus, severe remodeling of phospholipid metabolism is closely related to muscular dystrophy [36–39]. Laurila et al. found that inhibition of de novo sphingolipin synthesis counteracts muscular dystrophy [40]. Therefore, inhibition of sphingolipid synthesis may be a strong candidate for the treatment of muscular dystrophy.

Our results also found an association between the gut microbiota and DMD. Plasma levels of phenylacetylglutamine (PAGln) were increased. PAGln is a novel metabolic derivative of gut microbiota associated with cardiovascular diseases [41]. Myocardial involvement is common in patients with DMD, and plasma PAGln levels are associated with increased risks of cardiovascular disease and major adverse cardiovascular events (myocardial infarction, stroke or heart failure) [41]. In whole blood, isolated platelets, and animal arterial injury models, PAGln, has been shown to enhance platelet aggregation and thrombosis potential [41, 42]. In addition, the serum levels of hippuric acid in DMD decreased. Hippurate, one of the most abundant microbial-host co-metabolites, is formed in the liver and kidney during phase 2 detoxification by conjugating glycine and microbial benzoate [43]. We hypothesized that intestinal microbiota disorders in patients with DMD would lead to changes in hippuric acid levels. Moreover, the plasma levels of bile acids were disturbed. Besides regulating cholesterol, triglycerides, and fat-soluble vitamins digestion and absorption, bile acids also function as signaling molecules, which modulate epithelial cell proliferation, gene expression, and lipid and glucose metabolism via the activation of farnesoid X receptor (FXR) and G-protein-coupled bile acid receptor-1 (GPBAR-1, also known as TGR5) in the liver, intestine, muscle, and brown adipose tissue [44]. In DMD, levels of the bile acid glycoursodeoxycholic acid (GUDCA) are elevated. GUDCA is an antagonist of the bile acid receptor that increases hepatic bile acid levels. More importantly, deoxycholic acid-induced dysregulation of intestinal biology destroys cholic acid-enterohepatic circulation and promotes intestinal inflammation [45]. Deoxycholic acid-induced intestinal ecological disturbances may be the key etiology of intestinal inflammation, which is related to disturbances in bile acid metabolism and downregulation of the ileal FXR-fibroblast growth factor (FGF) 15 axis [45]. Studies have shown that FXR-FGF15/19 signaling affects skeletal muscle function as a symbiotic regulator of gut microbiota [46, 47]. Using fexaramine, a FXR agonist specific to the intestines, skeletal muscle mass and muscle performance were improved in aged mice [47]. FGF15/19-dependent ileal FXR signaling contributes to the increase in skeletal muscle protein synthesis, suggesting that it may be a potential therapeutic target for sarcopenia [46, 47]. These results also suggest that the intestinal FXR-FGF15/19 signaling pathway may play an important role in DMD, but its mechanism must be further explored.

Two recently published articles reported the role of intestinal microbia in DMD [48, 49]. Kalkan et al. showed that the gut microbiota composition differed significantly between wildtype and mdx mice, and the use of a metabolically supporting anti-inflammatory drug could revert the microbiome composition in the mutant mice [48]. The study of Farini et al. provided that, by using broad-spectrum antibiotics treatment in mdx mice, gut microbes were depleted, accompanied by a reduction in chronic muscle inflammation, thus additionally linking the dysbiotic microbiome composition and the phenotypic symptoms observed in mdx mice [49]. Given that gastrointestinal and metabolic issues are common in patients with DMD, these two studies unfold the possibility of using gut microbiota metabolite intervention strategies for managing or reversing DMD symptoms. In this study, we confirmed that metabolites of intestinal flora are disordered in DMD patients, provided support for treatment of intestinal microbia disturbance in DMD diseases.

## Conclusions

Overall, our study demonstrated the abnormal metabolism of amino acids, energy, and lipids in patients with DMD, consistent with pathological features, such as recurrent muscle necrosis and regeneration, interstitial fibrosis, and fat replacement. In addition, we also identified a number of differential metabolites associated with gut microbiota, which may be related to nutritional disorders and intestinal muscle dysfunction in DMD patients. Although our study provides a new research strategy for the pathogenesis of DMD, there are some limitations. First, the sample size was small, so we hope to conduct a multi-center study with a large sample size in a later stage to reduce sampling error. Second, due to the different types, treatment courses, and doses of corticosteroids used by DMD group in this article, we were unable to completely distinguish the corticosteroid-treated group from the untreated group using PCA. Therefore, we could not obtain differences in disease metabolism at corticosteroid treated or nontreated conditions. Although we cannot separate DMD patients into treated and untreated group, this article can still be considered as the first exploratory study on metabolic changes in clinical patients with DMD (regardless of medication use) in natural research history. In the future, we will conduct a prospective study with larger samples to focus on drug treatments (such as glucocorticoids, calcium channel blockers and vitamin D) and explore their impacts on the metabolic spectrum of DMD patients. Furthermore, target validation should be applied in an in-depth study to validate our selected metabolic indicators.

## Data Availability

The data that support the findings of this study are available on request from the corresponding author. The data are not publicly available due to the restriction that their containing information could compromise the privacy of research participants.

## List of abbreviations

DMD: Duchenne muscular dystrophy
HC: healthy control
UPLC-MS/MS: ultra-high performance liquid chromatography-tandem mass spectrometry
IRB: Institutional Review Board
QC: quality control
UPLC: ultra-high performance liquid chromatography
IDA: independent data acquisition
KEGG: Kyoto Encyclopedia of Genes and Genomes
HMDB: Human Metabolome Database
PCA: principal component analysis
PLS-DA: partial least-squares discriminant analysis
VIP: variable importance in projection
ANOVA: analysis of variance
LDS: least significant difference
PC: phosphatidylcholines
LPE: lysophosphatidylethanolamines
SM: sphingomyelin
FDR: false discovery rate
GLS: glutaminases
LPC: lysophosphatidylcholine
PA: phosphatidic acid
PS: phosphatidylserine
PAGln: phenylacetylglutamine
FXR: farnesoid X receptor
GPBAR-1: G-protein-coupled bile acid receptor-1
GUDCA: glycoursodeoxycholic acid
FGF: fibroblast growth factor
